# Supporting dataset and methods for serum concentrations of selected persistent organic pollutants measured in women with primary ovarian insufficiency

**DOI:** 10.1016/j.dib.2019.104430

**Published:** 2019-08-26

**Authors:** Wuye Pan, Shanshan Yin, Xiaoqing Ye, Xiaochen Ma, Chunming Li, Jianhong Zhou, Weiping Liu, Jing Liu

**Affiliations:** a MOE Key Laboratory of Environmental Remediation and Ecosystem Health, College of Environmental and Resource Sciences, Zhejiang University, Hangzhou, 310058, China; b Women's Reproductive Health Key Laboratory of Zhejiang Province, Women's Hospital, School of Medicine, Zhejiang University, Hangzhou, 310006, China; c College of Medical Technology, Zhejiang Chinese Medical University, Hangzhou, 310053, China

**Keywords:** Persistent organic pollutants, Polychlorinated biphenyls, Organochlorine pesticides, Primary ovarian insufficiency, Pretreatment methods

## Abstract

The dataset presented in this article supports “Selected persistent organic pollutants associated with the risk of primary ovarian insufficiency in women” (Pan et al., 2019). The supplementary data were as follows: (1) Detailed information regarding pretreatment methods, instrumental analysis and methods validation of quantification of serum concentrations of persistent organic pollutants (POPs). (2) The total dioxin equivalents (TEQs) levels of dioxin-like PCBs (DL-PCBs) in primary ovarian insufficiency (POI) cases and controls, as well as the association of TEQ levels with the risk of POI. (3) The results of principal components analyses (PCA) about 20 POPs that were detected in >40% samples.

**Table:** Specifications Table

Subject area	*Chemistry*
More specific subject area	*Analytical chemistry*
Type of data	*Tables and figures*
How data was acquired	*Gas chromatography-triple quadrupole mass spectrometer (GC-MS/MS) (Agilent 7890B GC/7000C)*
Data format	*Raw and Analyzed*
Experimental factors	*Spiked 0.3 mL of serum sample in a centrifuge tube with internal standards [PCB 209, tetrachloro-m-xylene (TCMX), isotopically labeled standards of PBDEs]. After three times of liquid-liquid extraction by extractant of n-hexane and dichloromethane (DCM) (1:1, v/v), evaporating the extracts to about 1 mL, and cleaned by a column filled with activated silica gel and Na*_*2*_*SO*_*4*_*. The elution was evaporated to dryness and redissolved in 50 μL of n-decane.*
Experimental features	*Recruited 157 primary ovarian insufficiency (POI) cases and 217 healthy controls. Serum concentrations of polychlorinated biphenyls (PCBs), organochlorine pesticides (OCPs), polybrominated diphenyl ethers (PBDEs) were measured.*
Data source location	*Zhejiang, China*
Data accessibility	*The data are given in this article*
Related research article	*Pan, W.; Ye, X.; Yin, S.; Ma, X.; Li, C.; Zhou, J.; Liu, W.; Liu, J. Selected persistent organic pollutants associated with the risk of primary ovarian insufficiency in women. Environment international. 129 (2019) 51–58*[1]

**Table:** 

**Value of the data** • The data in this article present information on the sample pretreatment method, instrumental analysis and method validation for determination of persistent organic pollutants (POPs) in serum samples. These data provide a reference for other scientists to optimize and validate pretreatment and quantification methods in human biomonitoring studies of POPs. • The data provide information on the distributions of the total dioxin equivalents (TEQs) levels of DL-PCBs in primary ovarian insufficiency (POI) cases and controls in China, which are complementary to the article of Pan et al. These data can be used to compare TEQ levels among different populations. • The PCA data are useful for understanding the multiple effects of exposure to mixtures of POPs.

## 1 Data

**Fig. 1:**
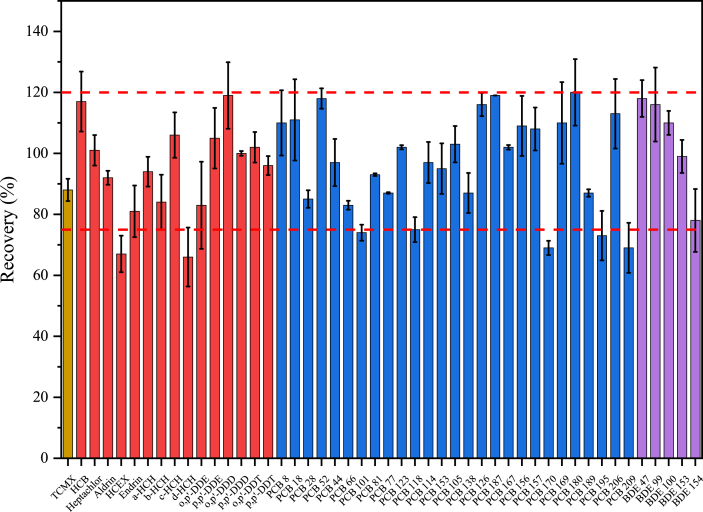
The average overall recovery of the analytes.

**Fig. 2:**
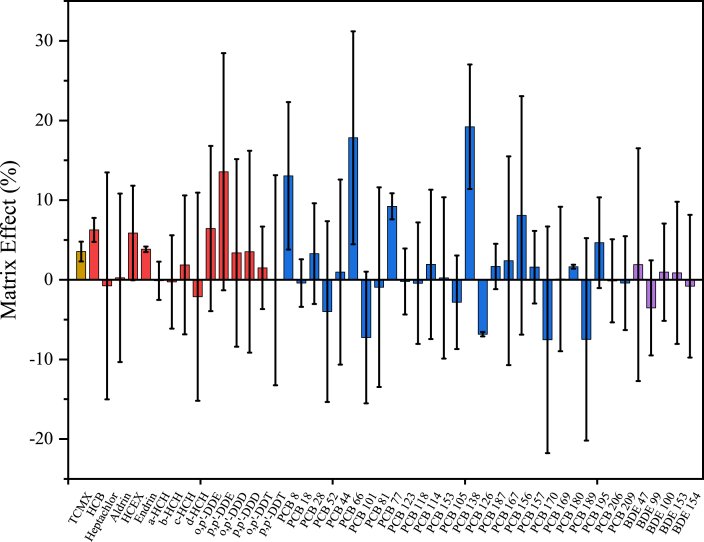
The matrix effects of the analytes.

The data reported here constitute the basis for the article by Pan et al. [1] The detailed information about sample pretreatment method, instrumental analysis and method validation for determination of persistent organic pollutants (POPs) in serum samples were presented in Table 1, Table 2, Table 3, Table 4, Table 5 and Fig. 1, Fig. 2. Table 6 and Table 7 showed the total dioxin equivalents (TEQs) levels of dioxin-like PCBs (DL-PCBs) in primary ovarian insufficiency (POI) cases and heathy controls, as well as the association of TEQ levels with the risk of POI. Principal components analyses results about 20 POPs that were detected in >40% samples were summarized in Table 8 and Table 9. The raw data of Table 2, Table 3, Table 6 were available in the file Supplementary Table 1, 2 and 4, respectively. The raw data of Fig. 1, Fig. 2 were available in the file Supplementary Table 3.

**Table 1:** Instrumental and quantification methods.

Type	Compound	IS	Quantifier			Qualifier			RT
			P	F	CE	P	F	CE	(min)
IS	TCMX		244	209	15	136	75.2	15	10.8
AT	PCB8	TCMX	222	152	30	152.1	151	5	11.7
AT	α-HCH	TCMX	216.9	181	5	181	145	5	11.7
AT	HCB	TCMX	283.9	213.9	35	283.9	248.8	25	11.9
AT	β-HCH	TCMX	216.9	181	5	181	145	5	12.3
AT	γ-HCH	TCMX	216.9	181	5	181	145	5	12.4
AT	PCB18	TCMX	256	186	30	258	186.1	30	12.6
AT	δ-HCH	TCMX	216.9	181	5	181	145	5	12.9
AT	PCB28	TCMX	256	186	30	258	186.1	30	13.6
AT	Heptachlor	TCMX	272	237	25	272	117	40	13.9
AT	PCB52	TCMX	292	220	35	222	150	35	14.3
AT	Aldrin	TCMX	262.9	192.9	40	262.9	190.9	40	14.6
AT	PCB44	TCMX	292	220	35	222	150	35	14.7
AT	HCEX	TCMX	352.9	262.8	25	352.9	281.9	20	15.5
AT	PCB66	TCMX	292	220	35	220	150	35	15.6
AT	o,p'-DDE	TCMX	246	176.2	30	248	176.2	30	16.1
AT	PCB101	TCMX	326	255.9	35	256	183.9	35	16.2
AT	PCB81	TCMX	292	220	35	222	150	35	16.8
AT	p,p'-DDE	TCMX	246	176.2	30	248	176.2	30	16.8
AT	PCB77	TCMX	292	220	35	222	150	35	17.0
AT	o,p'-DDD	TCMX	235	165.2	30	237	165.2	20	17.0
AT	Endrin	TCMX	262.9	192.9	35	262.9	190.9	35	17.4
AT	PCB123	TCMX	326	255.9	35	256	183.9	35	17.5
AT	PCB118	TCMX	326	255.9	35	256	183.9	35	17.6
AT	p,p'-DDD	TCMX	235	165.2	30	237	165.2	20	17.8
AT	PCB114	TCMX	326	255.9	35	256	183.9	35	17.9
AT	o,p'-DDT	TCMX	235	165.2	30	237	165.2	20	17.9
AT	PCB153	PCB209	360	289.9	30	290	218	30	18.2
AT	PCB105	TCMX	326	255.9	35	256	183.9	35	18.3
AT	p,p'-DDT	TCMX	235	165.2	30	237	165.2	20	18.8
AT	PCB138	PCB209	360	289.9	30	290	218	30	18.9
AT	PCB126	TCMX	326	255.9	35	256	183.9	35	19.2
AT	PCB187	PCB209	394	324	30	324	254	30	19.4
AT	PCB167	PCB209	360	289.9	30	290	218	30	19.7
AT	PCB156	PCB209	360	289.9	30	290	218	30	20.3
AT	PCB157	PCB209	360	289.9	30	290	218	30	20.5
AT	PCB170	PCB209	394	324	30	324	254	30	20.8
IS	BDE47		498	338	20	496	336	30	20.9
AT	BDE47	BDE47	486	326	20	484	324	30	20.9
AT	PCB169	PCB209	360	290	30	290	218	30	21.4
AT	PCB180	PCB209	394	324	30	324	254	30	21.7
AT	PCB189	PCB209	394	324	30	324	254	30	22.5
AT	PCB195	PCB209	430	360	30	358	288	30	22.9
IS	BDE99		576	416	20	576	418	20	23.5
AT	BDE99	BDE99	564	404	20	564	406	20	23.5
IS	BDE100		576	416	20	576	418	20	24.3
AT	BDE100	BDE100	564	404	20	564	406	20	24.3
AT	PCB206	PCB209	464	392	25	392	322	35	24.7
IS	PCB209		498	427	30	214	178	20	25.6
IS	BDE153		656	496	20	496	387	40	26.4
AT	BDE153	BDE153	644	484	20	484	375	40	26.4
IS	BDE154		656	496	20	496	387	40	27.5
AT	BDE154	BDE154	644	484	20	484	375	40	27.5

AT: Analytical Target compound, IS: internal standard. RT: retention time, P: Parent ion (*m*/*z*). F: Fragment ion (*m*/*z*).

CE: Collision Energy (eV).

**Table 2:** The accuracy and precision methods of PCBs.

Compound	Spiking levels	Blank Matrix				Within-run precision for serum from random donors (n = 3, RSD %)		
		Accuracy (Bias %)		Precision (RSD %)				
		Within-run	Between-run	Within-run	Between-run			
PCB8	Low	1.3%	4.4%	1.2%	3.8%	11.70%	4.65%	2.40%
	High	0.2%	2.7%	4.8%	10.5%			
PCB18	Low	3.4%	11.4%	6.9%	8.6%	6.00%	8.10%	2.10%
	High	1.9%	6.5%	2.5%	11.3%			
PCB28	Low	1.1%	6.8%	6.9%	11.6%	7.50%	0.90%	2.25%
	High	1.8%	3.0%	0.3%	11.0%			
PCB44	Low	6.4%	7.2%	7.1%	15.6%	8.10%	8.10%	6.15%
	High	4.6%	5.6%	6.8%	10.7%			
PCB52	Low	2.3%	4.2%	3.8%	6.9%	5.12%	4.56%	7.33%
	High	1.2%	5.0%	2.9%	4.1%			
PCB66	Low	8.0%	11.4%	9.5%	10.4%	4.95%	3.90%	8.55%
	High	5.1%	9.0%	6.0%	8.1%			
PCB101	Low	2.2%	15.0%	4.2%	5.4%	1.50%	1.20%	2.70%
	High	6.7%	8.0%	5.9%	9.9%			
PCB81	Low	9.2%	10.0%	2.1%	2.4%	4.80%	5.40%	6.15%
	High	6.8%	11.1%	2.8%	8.4%			
PCB77	Low	9.9%	10.0%	3.0%	7.0%	10.95%	11.70%	8.85%
	High	2.1%	5.0%	3.8%	8.7%			
PCB123	Low	0.3%	7.0%	0.5%	1.4%	3.00%	8.40%	5.10%
	High	9.6%	10.5%	0.3%	5.1%			
PCB118	Low	11.3%	13.4%	0.3%	2.2%	9.90%	10.65%	7.50%
	High	0.2%	6.8%	5.6%	10.1%			
PCB114	Low	5.4%	9.6%	0.5%	4.6%	9.00%	5.25%	2.40%
	High	3.1%	3.6%	10.5%	11.6%			
PCB153	Low	1.3%	14.2%	3.6%	10.2%	4.20%	11.70%	6.45%
	High	0.9%	11.7%	5.3%	10.7%			
PCB105	Low	1.7%	11.2%	4.8%	9.8%	10.35%	4.50%	10.65%
	High	5.7%	6.8%	4.1%	5.1%			
PCB138	Low	0.7%	1.4%	2.1%	12.4%	8.70%	9.75%	4.20%
	High	0.8%	1.4%	5.1%	7.5%			
PCB126	Low	7.2%	9.4%	7.6%	13.2%	6.90%	4.20%	1.95%
	High	1.5%	2.1%	4.1%	5.0%			
PCB187	Low	7.2%	14.6%	9.8%	13.2%	4.20%	8.70%	1.80%
	High	4.4%	4.5%	2.4%	7.4%			
PCB167	Low	4.3%	8.2%	1.1%	9.4%	5.10%	10.65%	6.90%
	High	4.1%	10.5%	6.0%	6.3%			
PCB156	Low	0.1%	3.2%	1.7%	6.4%	0.75%	3.75%	8.70%
	High	0.9%	6.9%	0.2%	6.3%			
PCB157	Low	7.7%	10.6%	6.6%	11.0%	5.70%	6.90%	6.15%
	High	6.5%	8.3%	1.8%	8.0%			
PCB170	Low	6.0%	10.4%	1.7%	2.0%	3.60%	5.10%	2.70%
	High	0.4%	3.6%	2.1%	9.6%			
PCB169	Low	2.1%	8.4%	4.8%	7.6%	8.25%	7.05%	7.65%
	High	1.7%	9.3%	2.6%	11.0%			
PCB180	Low	1.9%	2.0%	5.2%	12.8%	7.80%	6.30%	7.05%
	High	3.2%	6.8%	1.4%	3.6%			
PCB189	Low	3.6%	3.8%	5.8%	13.4%	2.70%	0.90%	3.75%
	High	2.2%	2.6%	6.8%	10.4%			
PCB195	Low	3.1%	4.0%	3.5%	8.6%	9.75%	10.80%	11.40%
	High	3.4%	9.9%	2.7%	5.1%			
PCB206	Low	0.1%	12.4%	1.8%	4.8%	6.75%	4.50%	10.80%
	High	0.0%	3.3%	4.8%	5.9%

**Table 3:** The accuracy and precision methods of OCPs and PBDEs.

Compound	Spiking levels	Blank Matrix				Within-run precision for serum from random donors (n = 3, RSD%)		
		Accuracy (Bias%)		Precision (RSD%)				
		Within-run	Between-run	Within-run	Between-run			
α-HCH	Low	0.6%	4.4%	4.0%	15.2%	6.60%	8.25%	6.30%
	High	1.7%	2.1%	5.4%	6.0%			
HCB	Low	2.0%	12.4%	3.0%	7.8%	9.00%	3.45%	11.55%
	High	5.9%	11.3%	2.9%	11.4%			
β-HCH	Low	13.8%	15.4%	1.6%	13.0%	7.05%	9.90%	1.95%
	High	0.3%	3.6%	3.3%	3.6%			
γ-HCH	Low	2.5%	10.2%	4.4%	6.8%	4.95%	8.40%	4.95%
	High	0.9%	1.4%	5.2%	11.1%			
δ-HCH	Low	2.1%	11.8%	4.2%	12.4%	5.68%	8.63%	4.58%
	High	1.5%	7.9%	3.1%	5.7%			
Heptachlor	Low	8.0%	11.2%	4.2%	9.4%	6.75%	1.20%	10.95%
	High	1.7%	5.3%	3.3%	6.9%			
Aldrin	Low	8.2%	15.6%	3.5%	6.6%	3.75%	3.15%	2.55%
	High	0.7%	8.3%	0.9%	1.4%			
HCEX	Low	8.7%	14.6%	5.2%	8.6%	9.00%	10.95%	7.35%
	High	4.9%	11.7%	1.0%	6.6%			
o,p'-DDE	Low	4.3%	10.6%	3.3%	6.8%	4.95%	11.25%	9.15%
	High	0.3%	1.5%	3.6%	10.5%			
p,p'-DDE	Low	1.5%	6.0%	3.4%	6.6%	2.40%	4.05%	10.80%
	High	6.9%	9.6%	1.5%	3.3%			
o,p'-DDD	Low	13.5%	14.0%	1.2%	2.4%	4.50%	3.75%	4.50%
	High	3.6%	9.8%	4.7%	10.7%			
Endrin	Low	0.4%	3.0%	3.9%	12.8%	10.05%	2.55%	2.85%
	High	4.8%	6.2%	5.2%	11.4%			
p,p'-DDD	Low	6.4%	15.2%	8.7%	14.6%	9.30%	5.85%	6.45%
	High	7.0%	7.4%	0.2%	4.5%			
o,p'-DDT	Low	0.9%	1.4%	1.8%	2.0%	0.75%	3.75%	11.40%
	High	1.4%	5.4%	2.4%	11.3%			
p,p'-DDT	Low	1.3%	4.2%	2.3%	3.2%	3.15%	1.20%	6.75%
	High	5.3%	8.9%	5.0%	11.7%			
BDE47	Low	3.6%	14.6%	0.7%	1.0%	11.40%	8.10%	10.05%
	High	3.1%	6.0%	0.4%	3.5%			
BDE99	Low	2.1%	2.6%	0.8%	1.8%	7.95%	9.30%	8.10%
	High	0.1%	1.7%	0.6%	6.6%			
BDE100	Low	1.2%	7.4%	4.1%	13.8%	4.20%	6.15%	1.50%
	High	6.4%	8.7%	5.1%	5.7%			
BDE153	Low	3.9%	7.2%	2.1%	4.8%	10.50%	3.90%	11.70%
	High	3.0%	3.8%	1.3%	4.7%			
BDE154	Low	9.3%	10.8%	1.4%	2.6%	1.35%	5.10%	11.40%
	High	1.8%	4.1%	8.8%	9.0%

**Table 4:** The calibration of POPs.

Compound	Calibration Curve	R^2^	Compound	Calibration Curve	R^2^
TCMX	y = 5134.48x	0.9996	o,p'-DDT	y = 11368.51x	0.9990
PCB8	y = 21697.78x	0.9995	PCB153	y = 11887.47x	0.9992
α-HCH	y = 3236.73x	0.9998	PCB105	y = 7448.54x	0.9956
HCB	y = 6203.90x	0.9996	p,p'-DDT	y = 8156.91x	0.9971
β-HCH	y = 2133.68x	0.9993	PCB138	y = 5233.74x	0.9987
γ-HCH	y = 2585.35x	0.9976	PCB126	y = 6742.35x	0.9985
PCB18	y = 14785.66x	0.9995	PCB187	y = 4682.77x	0.9996
δ-HCH	y = 2006.67x	0.9975	PCB167	y = 11514.99x	0.9986
PCB28	y = 20311.56x	0.9994	PCB156	y = 5974.90x	0.9990
Heptachlor	y = 4481.43x	0.9994	PCB157	y = 6378.60x	0.9992
PCB52	y = 6847.47x	0.9994	PCB170	y = 4395.06x	0.9987
Aldrin	y = 1769.68x	0.9998	BDE47 (IS)	y = 2094.29x	0.9985
PCB44	y = 6168.34x	0.9987	BDE47	y = 2252.61x	0.9982
HCEX	y = 951.55x	0.9997	PCB169	y = 4938.22x	0.9997
PCB66	y = 9201.21x	0.9993	PCB180	y = 3991.75x	0.9992
o,p'-DDE	y = 11969.61x	0.9996	PCB189	y = 4780.02x	0.9987
PCB101	y = 6792.92x	0.9993	PCB195	y = 2569.60x	0.9984
PCB81	y = 7976.40x	0.9995	BDE99 (IS)	y = 1247.71x	0.9979
p,p'-DDE	y = 9262.12x	0.9996	BDE99	y = 1261.05x	0.9991
PCB77	y = 7984.81x	0.9981	BDE100 (IS)	y = 1440.93x	0.9986
o,p'-DDD	y = 14217.39x	0.9979	BDE100	y = 1335.26x	0.9981
Endrin	y = 885.24x	0.9994	PCB206	y = 1459.70x	0.9990
BDE28 (IS)	y = 2475.46x	0.9984	PCB209	y = 3790.42x	0.9994
PCB123	y = 7646.52x	0.9982	BDE153 (IS)	y = 515.91x	0.9944
PCB118	y = 8653.99x	0.9926	BDE153	y = 534.59x	0.9975
BDE28	y = 2423.97x	0.9979	BDE154 (IS)	y = 368.79x	0.9974
p,p'-DDD	y = 11923.37x	0.9967	BDE154	y = 347.93x	0.9960
PCB114	y = 6956.96x	0.9952

**Table 5:** Method detection limit of POPs.

Compound	MDL (pg/mL)	Compound	MDL (pg/mL)
TCMX	0.944	o,p'-DDT	19.7
PCB8	7.00	PCB153	2.43
α-HCH	14.1	PCB105	11.5
HCB	1.05	p,p'-DDT	10.0
β-HCH	2.41	PCB138	9.38
γ-HCH	1.10	PCB126	3.76
PCB18	6.38	PCB187	1.41
δ-HCH	3.85	PCB167	9.87
PCB28	7.69	PCB156	4.78
Heptachlor	0.385	PCB157	19.1
PCB52	7.22	PCB170	4.58
Aldrin	1.24	BDE47 (IS)	0.763
PCB44	12.1	BDE47	0.992
HCEX	4.81	PCB169	14.1
PCB66	12.9	PCB180	6.44
o,p'-DDE	21.0	PCB189	2.62
PCB101	4.85	PCB195	1.96
PCB81	8.36	BDE99 (IS)	2.98
p,p'-DDE	30.7	BDE99	3.17
PCB77	1.86	BDE100 (IS)	2.92
o,p'-DDD	11.9	BDE100	1.24
Endrin	22.3	PCB206	4.36
BDE28 (IS)	0.923	PCB209	3.03
PCB123	14.7	BDE153 (IS)	25.6
PCB118	8.86	BDE153	17.7
BDE28	3.48	BDE154 (IS)	4.62
p,p'-DDD	5.42	BDE154	9.36
PCB114	1.43

**Table 6:** The TEQ levels of DL-PCBs in POI Cases and Controls.

DL-PCBs (pg/g lipid base)	Case		Control		*p*-Value^a^
	Median	IQR	Median	IQR	
PCB 77	0.90	0.07–1.39	0.09	0.03–0.98	<0.001
PCB 81	2.11	0.34–4.21	0.41	0.35–2.75	0.029
PCB 105	0.26	0.05–0.50	0.05	0.05–0.15	0.001
PCB 114	0.01	0.01–0.01	0.01	0.01–0.03	0.051
PCB 118	0.10	0.05–0.18	0.05	0.04–0.16	0.002
PCB 123	0.12	0.07–0.24	0.07	0.06–0.19	0.007
PCB 126	86.17	47.55–1108.55	58.49	50.24–132.37	0.003
PCB 156	0.02	0.02–0.03	0.02	0.02–0.03	0.643
PCB 157	0.08	0.07–0.09	0.08	0.07–0.09	0.204
PCB 167	0.04	0.04–0.05	0.04	0.04–0.05	0.112
PCB 169	51.97	60.01–67.30	62.71	54.03–69.16	0.216
PCB 189	0.01	0.01–0.04	0.01	0.01–0.01	0.147
∑_6_ DL-PCBs^b^	87.01	50.77–1116.93	63.52	53.84–135.09	0.005
∑_12_ DL-PCBs^c^	151.31	107.48–1178.15	130.71	113.12–218.40	0.005

IQR, Interquartile range.

a Mann-Whitney *U* test.

b ∑_6_ DL-PCBs includes PCB congeners 77, 81, 105, 118, 123, 126.

c ∑_12_ DL-PCBs includes PCB congeners 77, 81, 105, 114, 118, 123, 126, 156, 157, 167, 169, 189.

**Table 7:** Association of TEQ levels with POI in Binary Logistic Regression Models.

DL-PCBs	Unadjusted Model		Adjusted Model^a^	
	OR (95%CIs)	*p*-Value	OR (95%CIs)	*p*-Value
PCB 77	1.69 (1.35–2.12)	<0.001	1.84 (1.39–2.43)	<0.001
PCB 81	1.40 (1.13–1.73)	0.002	1.53 (1.18–1.99)	0.001
PCB 105	1.55 (1.24–1.93)	<0.001	1.88 (1.44–2.45)	<0.001
PCB 118	1.05 (0.85–1.29)	0.681	1.16 (0.90–1.50)	0.241
PCB 123	1.02 (0.83–1.26)	0.854	1.11 (0.85–1.43)	0.444
PCB 126	1.52 (1.22–1.89)	<0.001	1.75 (1.33–2.29)	<0.001
∑_6_ DL-PCBs^b^	1.50 (1.20–1.86)	<0.001	1.73 (1.32–2.26)	<0.001

a The adjusted model includes age, BMI, parity, history of breast-feeding, age at menarche, smoking, alcohol intake, education and annual household income.

b ∑_6_ DL-PCBs includes PCB congeners 77, 81, 105, 118, 123, 126.

**Table 8:** Total variance explained of principal components analysis.

Principle Component	Initial Eigenvalues			Extraction Sums of Squared Loadings			Rotation Sums of Squared Loadings		
	Total	% of Variance	Cumulative %	Total	% of Variance	Cumulative %	Total	% of Variance	Cumulative %
1	4.2	21.0	21.0	4.2	21.0	21.0	3.9	19.3	19.3
2	2.9	14.3	35.2	2.9	14.3	35.2	2.3	11.7	31.1
3	1.8	9.2	44.4	1.8	9.2	44.4	2.2	10.8	41.8
4	1.6	8.2	52.6	1.6	8.2	52.6	1.6	8.1	49.9
5	1.2	6.0	58.6	1.2	6.0	58.6	1.5	7.5	57.4
6	1.1	5.3	63.9	1.1	5.3	63.9	1.2	6.1	63.5
7	1.0	5.2	69.1	1.0	5.2	69.1	1.1	5.6	69.1
8	0.9	4.7	73.8						
9	0.9	4.6	78.4						
10	0.8	3.9	82.3						
11	0.7	3.5	85.8						
12	0.6	3.2	89.0						
13	0.6	2.8	91.8						
14	0.4	2.2	94.0						
15	0.4	2.0	95.9						
16	0.4	1.8	97.7						
17	0.2	1.2	98.9						
18	0.2	1.0	99.9						
19	0.0	0.1	100.0						
20	0.0	0.0	100.0

**Table 9:** Principal components analyses results.

Contaminant	PC-1 (21.0%)	PC-2 (14.3%)	PC-3 (9.2%)	PC-4 (8.2%)	PC-5 (6.0%)	PC-6 (5.3%)	PC-7 (5.2%)
PCB 8	−0.021	−0.036	0.185	0.070	**0.833**	0.120	0.000
PCB 18	0.025	**0.811**	0.098	0.122	0.167	−0.041	0.068
PCB 28	0.184	0.173	−0.103	**0.752**	0.035	0.149	0.122
PCB 52	**0.747**	0.101	−0.027	0.292	−0.054	0.137	0.042
PCB 77	−0.052	**0.685**	−0.185	−0.287	0.155	0.016	−0.090
PCB 81	**0.538**	0.471	−0.117	−0.128	0.352	−0.026	−0.175
PCB 105	0.170	0.012	−0.096	−0.048	−0.092	**0.484**	0.408
PCB 118	**0.979**	−0.052	−0.030	0.012	−0.019	0.025	0.047
PCB 123	**0.979**	0.004	−0.026	0.046	−0.021	0.076	0.055
PCB 126	−0.025	0.374	0.037	−0.065	**0.677**	−0.023	0.044
PCB 138	−0.012	**0.751**	0.355	0.183	−0.108	0.144	0.032
PCB 153	0.256	0.389	0.114	**0.465**	−0.140	0.469	0.110
PCB 187	**0.975**	−0.058	−0.029	0.002	−0.015	0.024	0.043
PCB 195	0.103	0.132	−0.027	0.326	0.025	−0.166	**0.487**
p,p'-DDT	−0.049	−0.256	−0.093	**0.677**	−0.002	−0.163	−0.180
p,p'-DDE	−0.045	0.006	**0.557**	−0.053	0.244	0.090	0.086
β-HCH	−0.041	0.050	**0.892**	−0.059	−0.094	−0.035	−0.083
γ-HCH	−0.027	0.087	**0.881**	−0.042	0.102	−0.038	−0.057
HCB	0.037	0.007	0.052	−0.005	0.204	**0.783**	−0.223
Heptachlor	−0.020	−0.071	0.003	−0.102	0.035	0.001	**0.732**

The bold means that the principal component has a high positive/negative loading for that contaminant.

## 2 Experimental design, materials and method

### 2.1 Optimized pretreatment

The target POPs in this study included polychlorinated biphenyls (PCBs), organochlorine pesticides (OCPs) and polybrominated diphenyl ethers (PBDEs). The pretreatment and analytical procedures were developed based on previous description with minor modification [2], [3]. A total of 0.3 mL of serum sample was spiked with 10 μL of mixture of internal standards (IS) [PCB 209, tetrachloro-m-xylene (TCMX), ^13^C_12_ isotopically labeled standards of PBDE 47, 99, 100, 153 and 154, 100 ng/mL]. Then, 0.5 mL of formic acid and 2.5 mL of ethanol were added and mixed. Ten milliliter of mixed extractant of n-hexane and dichloromethane (DCM) (1:1, v/v) was added. The mixture was ultrasonic extracted for 10 minutes and centrifuged at 2000 rpm for 10 minutes. The organic phase was transferred into a clean flat-bottomed flask. The extraction steps were repeated three times. The extracts were evaporated to about 1 mL and cleaned by a column filled with activated silica gel (6 g) and Na_2_SO_4_ (2 g). The column was eluted with 70 mL of a mixed solvent of n-hexane and DCM (1:1, v/v) before the addition of the concentrate. Then, the target compounds were eluted by another 70 mL of n-hexane and DCM (1:1, v/v). The elution was evaporated to dryness and redissolved in 50 μL of n-decane and stored in a refrigerator at 4 °C until quantification. All chemicals used above were purchased from J&K Chemical, Beijing, China.

### 2.2 Instrumental analysis

Gas chromatography-triple quadrupole mass spectrometry (GC-MS/MS) (Agilent 7890B GC/7000C) was used to quantitate the concentrations of POPs. The sample quantified methods were applied as described previously [2], [3]. For GC conditions, the column was DB-5ms (30 m × 0.25 mm × 0.25μm). Oven heating program was as follows: initial temperature at 80 °C hold for 1 min, and 10 °C/min to 180 °C hold for 5 min and then 20 °C/min to 220 °C (0 min) and finally 5 °C/min to 300 °C and hold for 5 min. The injector was kept at 250 °C. Carrier gas was helium (99.999% purity) at a constant flow rate of 1.0 mL/min. One microliter was splitlessly injected for each sample. The triplequad MS was operating in EI mode at 230 °C with electron ionization voltage of 70 eV and transfer line temperature at 280 °C. The multiple reaction monitoring mode was applied in the analysis process. For each analyte, two or more MRM transitions were monitored and one pair of ions with the highest peak area was chosen as the quantifier and the rest were set as qualifier. Detailed information is shown in Table 1. The quantification procedure was conducted using Agilent Masshunter Workstation Quantitative Analysis B.07.01 (Agilent Inc. Santa, Clara, CA, USA). The mass is set 0.9 or 0.1 for Agilent Workstation settings, recommended by the Agilent manual. The mass window is set at “UNIT” for both the first and second quadruple, which is 0.7 Å wide. For the retention time window, in the Agilent Masshunter, we set it at 1.0 min wide (−0.3 to +0.7) except for those with wider peaks.

### 2.3 Methods validation

A small-scale method validation was applied following the protocols established by the European Medicines Agency. Newborn bovine serum was used as the blank matrix. Calibration curves were analyzed in triplicates to estimate coefficients of determination (R^2^). Carryovers were assessed by injecting solvent blanks immediately after the analysis of the highest calibration point. Within- and between-run precision and accuracy of the methods were assessed over the course of three days using blank matrix spiked with target analytes at low (6 ng/mL of 10μL, final concentration of 0.2 ng/mL in the matrix) and high (300 ng/mL of 10μL, final concentration of 10 ng/mL in the matrix) concentrations and processed as described above. On each day, three replicates per spiking level, one blank matrix and one procedural blank were processed. All samples and blanks were spiked with IS (100 ng/mL of 10μL) prior to processing. Accuracy was calculated by subtracting the concentration measured in blank matrix from the concentration measured in low and high spiked samples. Precision and accuracy were considered satisfactory if results were <15% or <20% (for low spikes). Method detection limits (MDL) were determined using blank or low spiked blank matrix giving a signal-to-noise ratio (S/N) of 3. Recoveries of the extraction process were estimated using blank matrix spiked with native and mass labeled reference standards (at low and high concentrations) before and after extraction. Matrix effects were assessed by comparing the signal of reference standards in samples spiked after extraction with calibration standards prepared in n-decane. Background signals recorded in blank matrix samples were subtracted from analyte signals in post-extraction spikes prior to matrix effect calculation. Serum samples from three random different donors were extracted in triplicate to calculate the within-run precision using different matrices. These samples were only spiked at mid concentration.

### 2.4 Recovery and matrix effects

As shown in Fig. 1, the average overall recovery ranged between 78 and 113%, with relative standard deviations (RSDs) < 15% for all compounds.

Matrix effects were evaluated by comparing the signal of blank matrix spiking with native standards at low concentration (6 ng/mL of 10 μL, final concentration of 0.2 ng/mL in the matrix) or high concentration (300 ng/mL of 10 μL, final concentration of 10 ng/mL in the matrix) or IS (100 ng/mL of 10 μL) before and after extraction. In this study, corresponding IS was not available for some analytes, so matrix effects ranged from −20% to 35%, with RSDs below 15% for all compounds (Fig. 2).

### 2.5 Precision

For low spikes, the within- and between-run precision was lower than 20%, and for and high spikes, the precision was lower than 15% among three days for all target compounds. The inter-individual variation and the variation between the blank matrix and real human serum in precision of the method were assessed using serum samples from three random donors. The results showed the precision across different donors was acceptable (<15%) (Table 2, Table 3).

### 2.6 Accuracy

Low and high concentrations of target analytes were spiked into blank matrix. The nominal concentration in the guideline was defined as the sum of the background and spiked concentrations. However, as the POPs concentration in the blank matrix is lower than the MDL, the nominal concentration in this validation was set as the spiking concentration of the native standards. The accuracy for individual compounds was acceptable for all concentration levels (Bias <15%, or <20% for low spike) (Table 2, Table 3).

### 2.7 Calibration

Calibration curves were conducted using a mixture of native standards ranging from 0.1 ng/mL to 200 ng/mL and IS at concentration of 20 ng/mL in all calibrators. Calibration curves were computed using liner regression and were forced to pass zero. As shown in Table 4, coefficients of determination (R^2^) for all compounds were above 0.99.

### 2.8 Method detection limit (MDL)

Method detection limit (MDL) were estimated from low concentration standards giving a signal-to-noise ratio of 3 in the blank matrix. The MDL for this pretreatment process varied from 9 pg/mL to 173 pg/mL and 29 pg/mL and 575 pg/mL, respectively (Table 5).

### 2.9 Carry-overs

Solvent blanks (i.e. n-decane) were injected right after the highest concentration of calibration curve to assess carry-overs, which were below 20% of the MDL for all analytes. Overall, the results obtained during method validation indicate that the protocol is adapted for the analysis of targeted POPs. Thus, the method is suitable to be applied in the experiment.

### 2.10 Data analysis method

The TEQs were calculated by multiplying the toxic equivalence factors (TEFs) for each DL-PCB congener concentration: TEQ Σ_12_ DL-PCBs = PCB 77 × 0.0001 + PCB 81 × 0.0003 + PCB 105 × 0.00003 + PCB 1114 × 0.00003 + PCB 118 × 0.00003 + PCB 123 × 0.00003 + PCB 126 × 0.1 + PCB 156 × 0.00003 + PCB 157 × 0.00003 + PCB 167 × 0.00003 + PCB 169 × 0.03 + PCB 189 × 0.00003 [4]. Odds ratios (ORs) and 95% confidence intervals (CIs) for the risk of POI in association with TEQs levels were calculated by unconditional logistic regression models. The covariates included age, BMI, parity, history of breast-feeding, age at menarche, smoking, alcohol intake, education and annual household income [5], [6]. POPs concentration variables that were detected in >40% samples were subjected to principal components analysis (PCA) to produce a few number of summary PCA predictor variables. The data analysis were conducted using SPSS (version 20.0, IBM, Chicago, IL, USA).
